# Novel variants of HMW glutenin subunits from *Aegilops* section *Sitopsis* species in relation to evolution and wheat breeding

**DOI:** 10.1186/1471-2229-12-73

**Published:** 2012-05-30

**Authors:** Qian-Tao Jiang, Jian Ma, Yu-Ming Wei, Ya-Xi Liu, Xiu-Jin Lan, Shou-Fen Dai, Zhen-Xiang Lu, Shan Zhao, Quan-Zhi Zhao, You-Liang Zheng

**Affiliations:** 1 Triticeae Research Institute, Sichuan Agricultural University, Chengdu, Sichuan, 611130, China; 2 Lethbridge Research Centre, Agriculture and Agri-Food Canada, Lethbridge, T1J 4B1, Canada; 3 Key Laboratory of Southwestern Crop Germplasm Utilization, Ministry of Agriculture, Sichuan Agricultural University, Ya’an, Sichuan, 625014, China

## Abstract

**Background:**

High molecular weight glutenin subunits (HMW-GSs), encoded by the genes at *Glu-1* loci in wheat and its related species, are significant in the determination of grain processing quality. However, the diversity and variations of HMW-GSs are relatively low in bread wheat. More interests are now focused on wheat wild relatives in Triticeae. The genus *Aegilops* represents an important germplasm for novel HWM-GSs and other useful genes for wheat genetic improvement.

**Results:**

Six novel *Glu-1* alleles and HMW-GSs were identified and characterized from three species of *Aegilops* section *Sitopsis* (S genome). Both open reading frames (ORFs) and promoter regions of these *Glu-1* alleles were sequenced and characterized. The ORFs of *Sitopsis Glu-1* genes are approximately 2.9 kb and 2.3 kb for x-type and y-type subunits, respectively. Although the primary structures of *Sitopsis* HMW-GSs are similar to those of previously reported ones, all six x-type or y-type subunits have the large fragment insertions. Our comparative analyses of the deduced amino acid sequences verified that *Aegilops* section *Sitopsis* species encode novel HMW-GSs with their molecular weights larger than almost all other known HMW-GSs. The *Glu-1* promoter sequences share the high homology among S genome. Our phylogenetic analyses by both network and NJ tree indicated that there is a close phylogenetic evolutionary relationship of x-type and y-type subunit between S and D genome.

**Conclusions:**

The large molecular weight of HMW-GSs from S genome is a unique feature identified in this study. Such large subunits are resulted from the duplications of repetitive domains in *Sitopsis* HMW-GSs. The unequal crossover events are the most likely mechanism of variations in glutenin subunits. The S genome-encoded subunits, 1Dx2.2 and 1Dx2.2* have independent origins, although they share similar evolutionary mechanism. As HMW-GSs play a key role in wheat baking quality, these large *Sitopsis* glutenin subunits can be used as special genetic resources for wheat quality improvement.

## Background

High molecular weight glutenin subunits (HMW-GSs) are important storage proteins in seed endosperms of wheat and its related species [1,2]. Due to their composition effects on the elasticity of wheat dough, HMW-GSs play a significant role in the determination of grain processing quality [3]. HMW-GSs are encoded by the genes at *Glu-1* loci on the long arms of the Group 1 chromosomes (1A, 1B and 1D) in bread wheat. HMW-GSs can be further classified into two subfamilies (x-type and y-type), which are thought to have arisen from gene duplication events. Single copy of x-type and y-type gene occurs at two tightly linked loci, *Glu-1x* and *Glu-1y*. The HMW-GSs encoded by *Glu-1x* or *Glu-1y* can be distinguished from each other by the difference in their peptide lengths [1,4]. Previous studies indicated that allelic polymorphism in wheat HMW-GSs is associated with variations in the gluten viscoelasticity and bread making quality [1]. Up to now, a number of *Glu-1* alleles and HMW-GSs have been identified and characterized from wheat and its related species [5-18]. Sequence analyses of HMW-GS coding regions revealed that the primary structure of mature HMW-GSs consists of a central repetitive domain flanked by the conserved N-terminal and C-terminal regions [2]. The repetitive domain is mainly composed of repeat motifs including tripeptide, hexapeptide and nonapeptide. The difference among various HMW-GSs is mainly resulted from variable number of repeat motifs in the repetitive domains [2,19].

The section *Sitopsis* of genus *Aegilops* contains five species: *Aegilops bicornis*, (Forsskal) Jaub. & Spach. (S^b^S^b^, 2n = 2x = 14), *Ae*. *longissima* (Schweinf. & Muschl.) Á. Löve. (S^l^S^l^, 2n = 2x = 14), *Ae*. *sharonensis* (Eig) Á. Löve. (S^sh^S^sh^, 2n = 2x = 14), *Ae*. *searsii*, (Feldman & Kislev ex Hammer) Á. Löve. (S^s^S^s^, 2n = 2x = 14) and *Ae*. *speltoides*, (Tausch) Á.Löve, (SS, 2n = 2x = 14) [20]. Previous reports on cytogenetic and genetic investigations indicated that *Aegilops* genomes from this section of five species are closely related [21-24]. Although the characterization of HMW-GSs in two accessions of *Ae*. *searsii* have been reported [16], the *Glu-1* alleles and HMW-GSs in other four *Sitopsis* species have not been investigated. Our preliminary study surveyed the expression of HMW-GSs in *Ae*. *bicornisAe*. *longissima* and *Ae*. *sharonensis* and realized that the *Sitopsis Glu-1* alleles encodes the glutenin subunits with molecular weights much larger than other known HMW-GSs available in public databases. Here, we report the isolation and characterization of novel *Glu-1* alleles and HMW-GSs from *Ae*. *bicornisAe*. *longissima* and *Ae*. *sharonensis*. The objective of this study is to investigate the structural features of *Sitopsis* HMW-GSs, understand the evolutionary relationship of HWM-GS gene family within Triticeae, and further explore the potentials of S genome-encoded HMW-GSs in wheat quality breeding.

## Results

### Identification of *Aegilops* HMW-GSs and *Glu-1* alleles

**Figure 1:**
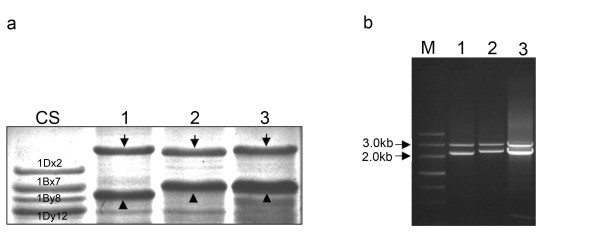
**Characterization of HMW-GSs isolated from*Aegilops*sect.*sitopsis*species.** (**a)** SDS-PAGE analysis revealed that both x-type and y-type HMW GSs (marked by arrow and triangle, respectively) are expressed in three *Aegilops* accessions and all six subunits have the molecular weights larger than those of Chinese Spring. (**b)** The complete DNA ORFs of both x-type and y-type HMW-GSs were amplified from S genome. Lane 1, *Ae*. *bicornis*; Lane 2, *Ae*. *longissima*; Lane 3, *Ae*. *sharonensis*; CS, Chinese Spring; M is the 1Kb DNA ladder.

The SDS-PAGE profiles on three accessions of *Ae*. *bicornisAe*. *longissima* and *Ae*. *sharonensis* indicated that *Sitopsis* HMW-GSs consist of large x-type and y-type subunits which migrate significantly slower than the same type of subunits in Chinese Spring (Figure 1a). Subsequent cloning of the *Glu-1* ORFs further verified that the molecular weights of these *Sitopsis* x-type subunits are close to or larger than that of 1Dx2.2, one of largest HMW-GSs previously reported [25]. The results of N-terminal sequencing suggested that the protein bands with slower and faster mobility are x- and y-type subunits, respectively. The obtained sequences of seed protein are perfectly matched to those deduced from the cloned genes (Table 1).

**Table 1:** Comparison of the *N*-terminal amino acid sequences derived from protein sequencing with those deduced from the cloned DNA sequences encoding for HMW-GS in three *Aegilops* species

**Species**	**Subunit**	**Residue**											
		1	2	3	4	5	6	7	8	9	10	11	12
*Triticum aestivum*	x-type consensus	**E**	**G**	**E**	**A**	**S**	**G/E**	**Q**	**L**	**Q**	**C**	**E**	**R/H**
*Ae*. *bicornis*	1S^b^x sequenced	E	G	E	A	S	G	Q	L	Q	C	E	R
	1S^b^x deduced	E	G	E	A	S	G	Q	L	Q	C	E	R
*Ae*. *longissima*	1S^l^x sequenced	E	G	E	A	S	G	Q	L	Q	C		
	1S^l^x deduced	E	G	E	A	S	G	Q	L	Q	C	E	R
*Ae. sharonensis*	1S^sh^x sequenced	E	G	E	A	S	G	Q	L	Q	C	E	
	1S^sh^x deduced	E	G	E	A	S	G	Q	L	Q	C	E	R
*T*. *aestivum*	y-type consensus	**E**	**G**	**E**	**A**	**S**	**R/K**	**Q**	**L**	**Q**	**C**	**E**	**R**
*Ae*. *bicornis*	1S^b^y sequenced	E	G	E	A	S	R	Q	L	Q	C	E	R
	1S^b^y deduced	E	G	E	A	S	R	Q	L	Q	C	E	R
*Ae*. *longissima*	1S^l^y sequenced	E	G	E	A	S	R	Q	L	Q	C	E	
	1S^l^y deduced	E	G	E	A	S	R	Q	L	Q	C	E	R
*Ae. sharonensis*	1S^sh^y sequenced	E	G	E	A	S	R	Q	L	Q	C	E	R
	1S^sh^y deduced	E	G	E	A	S	R	Q	L	Q	C	E	R

The consensus sequences of x-type and y-type HMW subunits of bread wheat were also list as standard. The results indicated that the amino acid sequences translated from the cloned genes are perfectly matched to those of the native protein of seeds.

The PCR amplicons of *Sitopsis Glu-1* alleles are composed of two DNA fragments (approximately 2.9 kb and 2.3 kb) for each of three accessions (Figure 1b). All amplified PCR products were cloned and the *Glu-1* ORFs at different alleles were determined by the sequence analysis and enzyme digestions. The full length of *Glu-1* ORFs was obtained by using the method of primer walking and nested deletion. Six sequences for x-type and y-type HMW-GSs from the S genome of three *Aegilops* species were designated as 1S^b^x2.9 and 1S^b^y2.3 (*Ae*. *bicornis*), 1S^l^x2.9 and 1S^l^y2.3 (*Ae*. *longissima*), 1S^sh^x2.9 and 1S^sh^y2.3 (*Ae*. *sharonensis*), respectively. All these DNA sequences have been deposited into the NCBI database with the Genbank accession numbers from JN001481 to JN001486.

### Expression of *1S*^*sh*^*x2.9* and *1S*^*sh*^*y2.3* in bacterial cells

**Figure 2:**
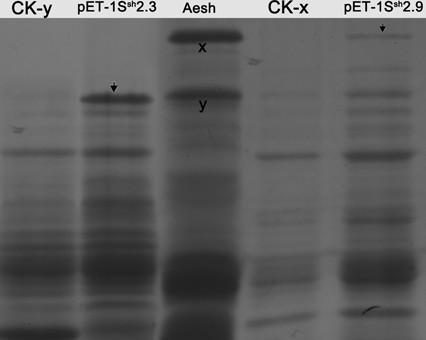
**Bacterial expression of the modified ORFs of two alleles*1S***^***sh***^***x2.9*and*1Sshy2.3*in*E. coli*BL21 (DE3) and SDS-PAGE analysis of expressed products.** The modified ORFs were prepared by removing the signal peptide sequence from each of the sequences by PCR mutagenesis. Protein extracts were prepared by dissolving cells directly in SDS-PAGE sample buffer. The glutenin proteins synthesized in *E. coli* directed by *1S*^*sh*^*x2.9* and *1Sshy2.3* under IPTG induction showed identical electrophoretic mobility to those from seeds of *Ae. sharonensis* (shown by arrows ). CK-x, y: proteins extracted from bacteria harbouring recombinant vectors pET–1S^sh^x2.9 or pET–1S^sh^y2.3 without IPTG induction for control; Aesh: proteins extracted from seeds of *Ae. sharonensis*.

After removing the coding sequence for the signal peptide from the ORFs of *1S*^*sh*^*x2.9* and *1S*^*sh*^*y2.3*, the modified ORFs were cloned into pET-30. Two bacterial expression constructs (pET-1S^sh^x2.9 and pET-1S^sh^y2.3) were chosen to express mature protein in bacterial cells. In the cells harboring pET-1S^sh^x2.9 and pET-1S^sh^y2.3, IPTG induction led to the expression of the protein bands with electrophoretic mobility similar to those of the native x and y-type subunits from the seed extract of *Ae*. *sharonensis* (Figure 2).

### Structural characteristics of primary sequences of *Aegilops* HMW-GSs

**Figure 3:**
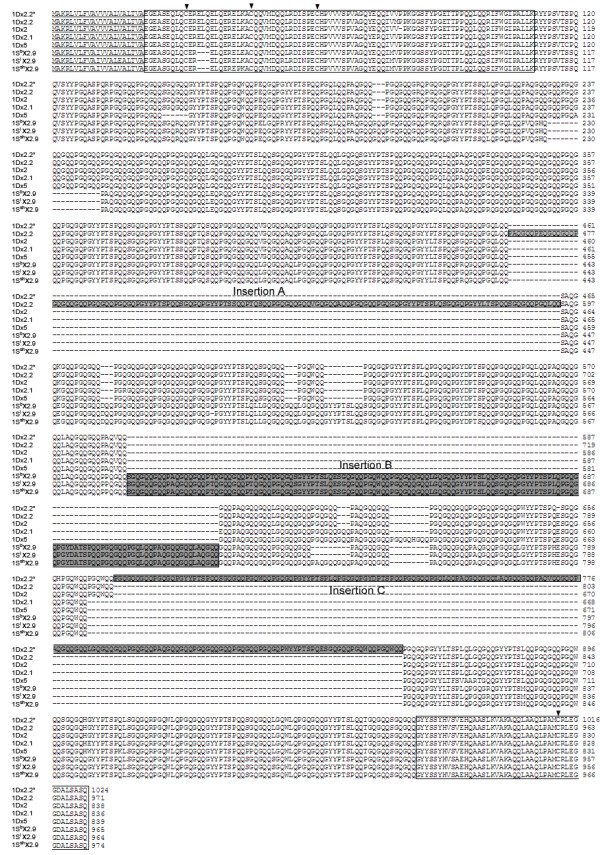
**Sequence comparison of x-type HMW-GSs isolated from D and S genomes.** The comparison of x-type subunits indicated that the inserted amino acid fragments in 1Dx2.2, 1Dx2.2* and 1Sx subunits (designated as Insertion **A**, **B** and **C**) are independent. Signal peptide is underlined; the N-terminal and C-terminal regions are boxed, respectively. The conserved cysteine residues are indicated by triangles.

**Figure 4:**
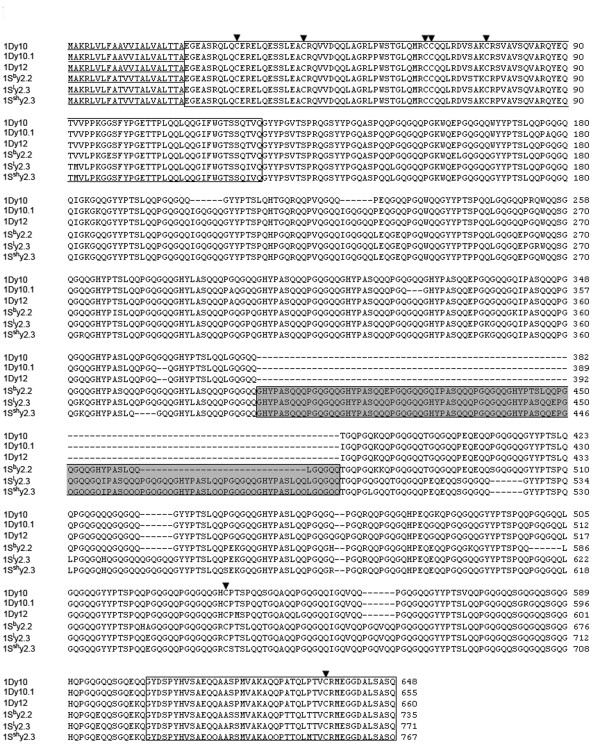
**Sequence comparison of y-type HMW-GSs isolated from D and S genomes.** The comparison of y-type subunits indicated that the insertions in 1Sy subunits have never been identified and characterized in other known subunits.

**Figure 5:**
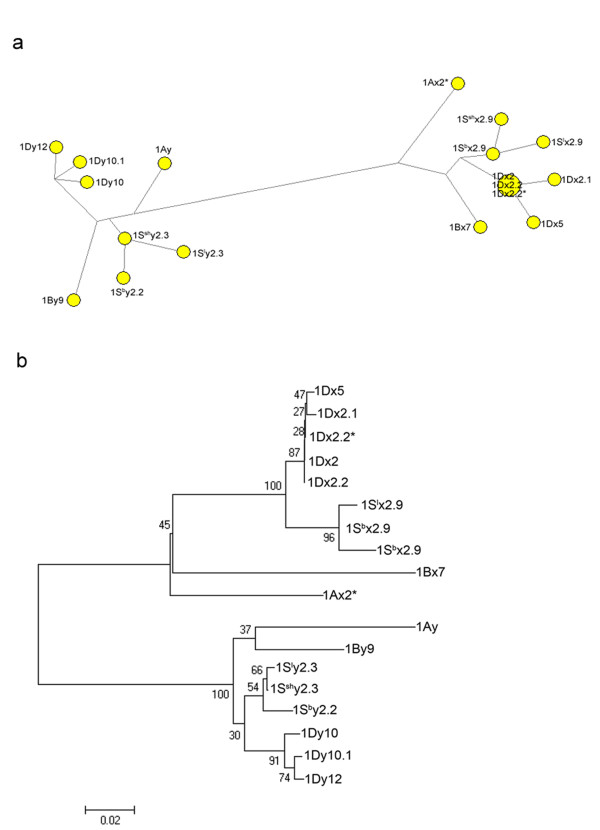
**Schematic diagram of primary structure of large HMW-GSs from D and S genomes.** The inserted fragments in **(a)** 1Dx2.2, **(b)** 1Dx2.2*, **(c)** 1Sx, and **(d)** 1Sy subunits are originated from independent duplication events. The new regions arose from block insertions are shown by the boxes and the length of each insertion is numbered in the box.

We predicted the amino acid sequences of six *Sitopsis* HMW-GSs and found that their primary structures are composed of four regions, i.e. a signal peptide, a central repetitive domain, the conserved N-terminal and C-terminal. The distribution and number of cysteine residues are identical to those in typical x-type and y-type subunits (Figure 3, 4; Table 2). The deduced protein sequences were firstly aligned with other known HMW-GSs from A, B and D genomes. Such comparison demonstrated that there is a higher similarity between S and D genome. Therefore, we realigned the HMW-GSs identified from three *Sitopsis* accessions in this study with those encoded by D genome available in public databases to determine their evolutionary relationship (Figure 3, 4). Our results indicated that the S genome-encoded glutenin subunits considerably differ from other known HMW-GSs. Compared to 1Dx2, *Sitopsis* x-type subunits (1S^b^x2.9, 1S^l^x2.9 and 1S^sh^x2.9) share the insertion of 141 residue with five tripeptides, 15 hexapeptides and four nonapeptides (Figure 5a-c). For *Sitopsis* y-type subunits, both 1S^l^y2.3 and 1S^sh^y2.3 have an insertion of 105 residues with seven hexapeptides and seven nonapeptides (Figure 5d), but this duplicated block in 1S^b^y2.3 only contains five hexapeptides and five nonapeptides (a total of 75 residues). We found that the peptide insertions in both x-type and y-type subunits from three *Sitopsis* species are copied from the adjacent regions, with some variations in single or more amino acid residues.

**Table 2:** Comparison of primary structures of HMW-GSs

	**Number of amino acid residues**				**Number of cysteine residues**			
	**N-terminal domain**	**Repetitive domain**	**C-terminal domain**	**Total**	**N-terminal domain**	**Repetitive domain**	**C-terminal domain**	**Total**
1Ax2*	86	666	42	794	3	0	1	4
1Bx7	81	645	42	768	3	0	1	4
1Dx2	88	687	42	817	3	0	1	4
1Dx2.1	89	984	42	815	3	0	1	4
1Dx5	89	687	42	818	3	1	1	5
1Dx2.2	89	**819**	42	**950**	3	0	1	4
1Dx2.2*	89	**872**	42	**1003**	3	0	1	4
1S^b^x2.9	86	**816**	42	**944**	3	0	1	4
1S^l^x2.9	86	816	42	**944**	3	0	1	4
1S^sh^x2.9	86	825	42	**953**	3	0	1	4
1Ay (Ta-e3)	104	**583**	42	**732**	5	1	1	7
1By9	104	538	42	684	5	1	1	7
1Dy10	104	481	42	627	5	1	1	7
1Dy12	104	493	42	639	5	1	1	7
1S^b^y2.3	104	**568**	42	**714**	5	1	1	7
1S^l^y2.3	104	**604**	42	**750**	5	1	1	7
1S^sh^y2.3	104	**600**	42	**746**	5	1	1	7

The numbers of amino acid residues in bold are for large subunits.

### Structural characteristics of 5’ flanking promoters of *Aegilops* HMW-GSs

The 5’ flanking promoter regions of HMW-GS genes in three *Sitopsis* species were amplified by using two pairs of PCR primers specific to x-type and y-type subunits, respectively. All amplified PCR products were cloned and sequenced. Based on previous studies, the promoter regulatory elements of HMW-GSs are composed of TATA box, complete and partial HMW enhancers, E and N motifs [26]. Our results indicated that the amplified promoter regions of *Sitopsis* HMW-GSs cover all recognized promoter regulatory elements. The DNA lengths of *1S*^*b*^*x2.9, 1S*^*l*^*x2.9* and *1S*^*sh*^*x2.9* promoter are identical (904 bp); whereas those of *1S*^*b*^*y2.31S*^*l*^*y2.3* and *1S*^*sh*^*y2.3* varied from 845 bp to 919 bp. The characterized promoter sequences of *Sitopsis* x-type and y-type HMW-GSs were aligned to homologous regions of 1Ax2*, 1Bx7, 1Dx2Triticum urartu 1Ay1By9 and *1Dy10*, respectively. Multiple sequence alignments showed that both types of glutenin subunits encode the conserved domains and variable parts in their promoter regions. We found that the HMW-GS promoters mainly differentiate with base substitutions, insertions, or deletions (data not shown). All the regulatory elements in the characterized *Sitopsis* HMW-GS promoters share the high conservation with few substitutions (Table 3). An 85 bp fragment, in which the partial HMW enhancer was included, was deleted in the 5’ flanking promoter regions of *1S*^*sh*^*y2*.*3* (Table 3). This deletion has not interrupted the expression of *1S*^*sh*^*y2*.*3*.

**Table 3:** Sequences variations of regulatory element among different HMW-GS promoters

**Alleles**	**E motif (TGTAACCC)**	**N motif (TGAGTCAT)**	**Partial Enhancer (TTTGCAAA)**	**Enhancer (GTTTTGCAAAGCTCCAATTGCTCCTTGCTT ATCCAGCT)**	**TATA box** **(CTATAAAAG)**	**Start** **(TTATCA)**
1Ax2*	TGTAA**AT**C	TGAGTCA**C**	TTTGCAAA	GTTTT**A** CAAAGCTCCAATTGCTCCTT GCTT ATCCAGCT	CTATAAAAG	T**CT**TCA
*1Bx7*	TGTAA**AT**C	TGAGTCAT	TTTGC**GG**A	GTTTTG C**-** AAGCTCCAATTGCTCCTT **A**CTT ATCCAGCT	CTATAAAAG	T**C**ATCA
*1Dx2*	TGTAA**A**CC	TGAGTCA**C**	TTTGCAAA	GTTTTG CAAAGCTCCAATTGCTCCTT GCTTATCCAGCT	CTATAAAAG	TTATCA
*1Dx5*	TGTAA**A**CC	TGAGTCA**C**	TTTGCAAA	GTTTTG CAAAGCTCCAATTGCTCCTT GCTTATCCAGCT	CTATAAAAG	TTATCA
*1S*^*b*^*x2.9*	TGTAA**AT**C	TGAGTCA**C**	TTTGCAAA	GTTTTG CAAAGCTCCAATTGCTCCTT **T**CTTAT**T**CAGCT	CTATAAAAG	T**C**ATCA
*1S*^*l*^*x2.9*	TGTAA**AT**C	TGAGTCA**C**	TTTGCAAA	GTTTTG CAAAGCTCCAATTGCTCCTT **T**CTTAT**T**CAGCT	CTATAAAAG	T**C**ATCA
*1S*^*sh*^*x2.9*	TGTAA**AT**C	TGAGTCA**C**	TTTGCAAA	GTTTTG CAAAGCTCCAATTGCTCCTT **T**CTTAT**T**CAGCT	CTATAAAAG	T**C**ATCA
*1Ay*	TGTAA**AT**C	**C**GAGTCAT	**deleted**	GTTTTG CAAAGCTCCAATTGCTCCTT GCTTATCCAGCT	CTATAAAAG	T**C**ATCA
*1By9*	TGTAA**AT**C	TGA**T**TCAT	T**C**T**A**CAAA	GTTTTG CAAA**A**CTCCAATTGCTCCTT GCTTATCCAGCT	CTATAAAAG	T**C**ATCA
*1Dy10*	TGTAA**AT**C	TGAGTCAT	TTTGCAAA	GTTTTG CAAAGCTCCAATTGCTCCTT GCTTATCCAGCT	CTATAAAAG	T**C**ATCA
*1S*^*b*^*y2.2*	TGTAA**A**CC	TGAGTCA**C**	TTTGCAAA	GTTTTG CAAAGCTCCAATTGCTCCTT GCTTATCCAGCT	CTATAAAAG	T**C**ATCA
*1S*^*l*^*y2.3*	TGTAA**AT**C	TGAGTCAT	TTTGCAAA	GTTTTG CAAAGCTCCAATTGCTCCTT GCTTATCCAGCT	CTATAAAAG	TTATCA
*1S*^*sh*^*y2.3*	TGTAA**AT**C	TGAGTCAT	**deleted**	GTTTTG CAAAGCTCCAATTGCTCCTT GCTTATCCAGCT	CTATAAAAG	T**C**ATCA

The position of each variation is underlined in the corresponding locus of consensus sequences.

### Phylogenetic relationship of HMW-GSs between S and other genomes

**Figure 6:**
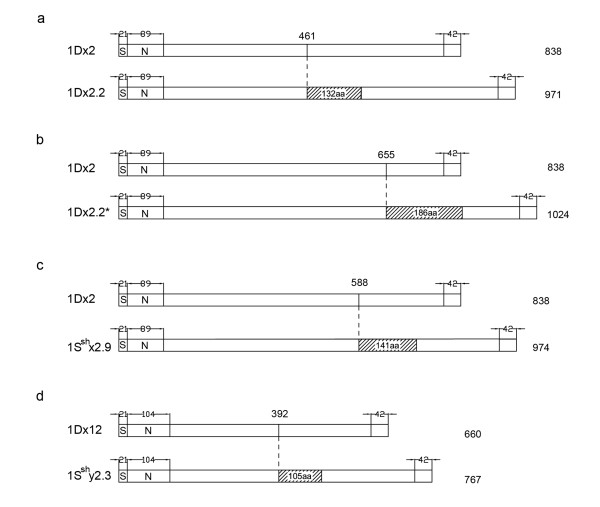
**Evolutionary relationship between HMW-GSs alleles of S genomes and those of*Glu-A1*,*Glu-B1*and*Glu- D1*.** Phylogenetic analysis was constructed from nucleic acid sequence variations of the 5’ flanking promoter sequences, plus the sequences encoding the signal peptides and N-terminal regions. Both the network (**a**) and the neighbor-joining (**b**) tree indicated that all HMW-GS alleles are clustered into two groups (x-type and y-type subunits) which strongly support the close relationship between S genome HMW-GS alleles and those of D genome.

To investigate the evolutionary relationship between S genome-encoded HMW-GS alleles and those of A, B, D genomes, we constructed the phylogeny of a network and a neighbor-joining tree (Figure 6a, b). The 5’ flanking promoter sequences, plus the sequences encoding signal peptides and the N-terminal, were selected for phylogenetic analysis because they have demonstrated to be phylogenetically informative. Firstly, our previous study on HMW-GS promoter indicated that the regulatory elements which control the tissue specificity and expression level of different HMW-GS genes are well conserved in diploid species of Triticeae [27]. Secondly, the sequences encoding signal peptides and N-terminal domain are also relative conserved. Therefore, these HMW-GS sequences were suitable for phylogenetic analysis [17,28].

Our network analysis demonstrated that all HMW-GS alleles are clustered into two groups (x-type and y-type subunits) (Figure 6a). Both x-type and y-type subunits showed a star-like phylogeny from principle nodes. In the x-type group, *1S*^*l*^*x2.9* and *1S*^*sh*^*x2.9* are linked to *1S*^*b*^*x2.9*, and then form a close link to the principle node which is composed of *1Dx2*, *1Dx2.2* and 1Dx2.2*. For the y-type group, *1S*^*b*^*y2.3*, *1S*^*l*^*y2.3* and *1S*^*sh*^*y2.3* are formed a parallel link to the y-type genes encoded by D genome. The resulted NJ tree also indicated that x-type and y-type subunits are divided into two clades which support a close phylogenetic relationship on HMW-GSs between S and D genome (Figure 6b). Therefore, our results from both network and neighbor-joining tree demonstrated that S genome-encoded HMW-GS alleles are evolutionally related to those of D genome.

## Discussion

As HMW-GSs play the key role in determining wheat gluten and dough elasticity, the characterization on novel HMW-GSs from Triticeae wild germplasm will be beneficial not only for improving wheat end-use quality but also for further understanding the structure variation and evolution of this important protein family. Compared to bread wheat, wild Triticeae grasses have more HMW-GS variants. For example, a number of HMW-GS variants with novel structural characteristics have been identified from *Aegilops* genus [16,18,29]. However, the progress on characterizing the HMW-GS expression from *Aegilops* section *Sitopsis* species remains slow and our knowledge on their structure, function, and evolution is still limited. In this study, we report the isolation and characterization of six x-type and y-type HMW-GSs variants from the S genome of three *Aegilops* species (*Ae*. *bicornisAe*. *longissima* and *Ae*. *sharonensis*). These novel variants will be useful to widen or enrich *Glu-1* genes and HMW-GSs for wheat quality breeding.

### Structural variations and evolution of *Glu-S1* alleles and possible mechanism

To avoid the potential error in PCR or sequencing, each nucleotide sequence was determined by multiple independent clones. The results of N-terminal sequencing indicated that the first 10 residues of N-terminal protein sequence of all 6 x and y-types subunits deduced from DNA sequences match perfectly to those directly determined by protein sequencing. And further bacterial expression proved that the cloned sequences are indeed accurate representations of the coding genes of HMW-GS in three *Aegilops* species. Therefore, the molecular information for *GluS1* alleles obtained in this study is reliable and suitable for exploring structural differentiation and evolution of *Glu-S1* alleles. Our results demonstrated that each of three *Aegilops* section *Sitopsis* species has two expressed subunits and the possession of large molecular weights is unique in both x-type and y-type subunit of S genomes (S^b^, S^l^ and S^sh^). Previous study reported that 1Dx2.2* and 1Dx2.2 are the largest HMW-GSs and their mature subunits contain 1003 and 950 amino acid residues, respectively [25,30]. In this study, we identified that the length of *Ae*. *sharonensis* subunit 1S^sh^x2.9 is 953 residues, shorter than 1Dx2.2* but longer than 1Dx2.2, which means 1S^sh^x2.9 is the second largest HMW-GSs characterized so far (Table 2). In addition, 1S^b^x2.9 of *Ae*. *bicornis* and 1S^l^x2.9 of *Ae*. *longissima* also have large molecular weights close to that of 1Dx2.2. For y-type HMW-GS genes, the lengths of their complete ORFs are usually less than 2 kb. Our previous study reported that a HMW-GS gene variant *1Ay (Ta-e3)*, isolated from einkorn wheat, encodes its ORF with the length of 2202 bp, larger than all other known y-type genes [17]. In this study, however, we identified two novel y-type *Glu-1* alleles, *1S*^*l*^*y2*.*3* from *Ae*. *longissima* and *1S*^*sh*^*y2*.*3* from *Ae*. *sharonensis*, and determined that their ORF lengths are 2256 and 2242 bp, respectively, much larger than that of *1Ay (Ta-e3)* (Table 2). As the y-type HMW-GS genes with such large molecular weights have not been reported in wheat and its related species, both *1S*^*l*^*y2.3* and *1S*^*sh*^*y2.3* will be special and useful to extend our knowledge on structure, function, evolution of the y-type HMW-GSs.

**Figure 7:**
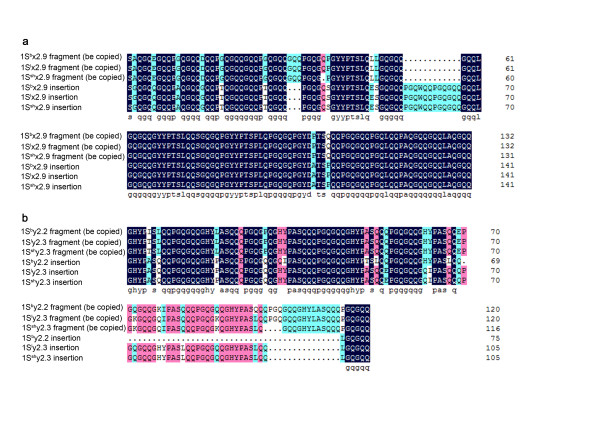
**Comparative analysis of amino acid sequences of HMW-GS repeated and inserted regions for 1Sx (a) and 1Sy (b) subunits.** The residues highlighted in black boxes represent complete sequence identities and the residues highlighted in gray boxes represent partial sequence identities.

Four modes have been proposed for the sequence alteration and evolution of HMW-GSs: (1) single residue changes, (2) deletion or insertion in a repeat unit, (3) single repeat changes, and (4) deletions or duplications of repeat blocks [6]. It has been reported that the unequal crossover events and slip-mismatching are the most likely mechanism of the size variations in HMW-GSs [25,31]. In this study, we found that the large molecular weights of S genome-encoded subunits are almost entirely due to the insertion and duplication of these repeat motifs (Figure 3,4). Previous study on comparative analysis of peptide sequences indicated that 1Dx2.2 and 1Dx2.2* are evolved from the two separate duplications of 132 and 186 residues, respectively [30]. Although three S genome-encoded x-type subunits, 1Dx2.2 and 1Dx2.2* have been resulted from duplication events, they are different in three ways. Firstly, each duplication of 1Sx subunits, 1Dx2.2 and 1Dx2.2* occur at different positions of repetitive domains (Figure 5a-d; Table 4). Secondly, the duplicated regions contain the varied numbers of repeat motifs which result in distinct size of inserted fragments among 1Sx subunits, 1Dx2.2 and 1Dx2.2*. Thirdly, the inserted fragments from duplications in 1Sx subunits are not completely identical to that from which it was duplicated (Figure 7). On the contrary, the new inserted regions in 1Dx2.2 and 1Dx2.2* are perfect copy of adjacent region without any changes. Based on above discussions, we can conclude that *Sitopsis* x-type subunits, 1Dx2.2 and 1Dx2.2* may have independent origins, although they share the similar evolutionary mode. We realized that such similar pattern also exists in the S genome-encoded y-type subunits.

**Table 4:** Repetitive motifs of the inserted fragments in repetitive domains of S genome-encoded HMW-GSs identified in this study and those of previously characterized one

**Subunit**	**Number of tripeptide**	**Number of hexapeptide**	**Number of nonapeptide**	**Total number of amino acid residues**
1Dx2.2	4	14	4	132
1Dx2.2*	8	21	4	186
1S^b^x2.9	5	15	4	141
1S^l^x2.9	5	15	4	141
1S^sh^x2.9	5	15	4	141
1S^b^y2.3	0	5	5	75
1S^l^y2.3	0	7	7	105
1S^sh^y2.3	0	7	7	105

### Implications of novel *Aegilops* HMW-GSs for wheat quality breeding

Two structural features of HMW-GSs may be relevant to their participation as gluten polymers in the baking quality of dough. Firstly, the number and distribution of cysteine residues determine the forming inter- and intra-molecular disulphide bonds. It is well known that disulphide bonds play a key role in determining the structure and properties of wheat glutenin polymers. The presence of an additional cysteine residue in the repetitive region of subunit 1Dx5 was reported to be responsible for the correlation of this particular HMW-GS with good bread-making quality [32,33]. We found that the S genome-encoded subunits have the conserved cysteine composition, which may be important to keep the normal gluten polymer. Secondly, the properties and interactions of repetitive domains are important in determining the dough viscoelastic properties [34]. The positive relationship between the HMW-GS sizes and their effects on dough strength has been revealed by previous studies. Belton [35] and Feeney et al. [36] proposed a model in which the gluten polymers interact via inter-chain hydrogen bonds between the subunit repetitive domains and more stable interactions can be formed with longer subunits. The experiments of incorporating the 1Dx2.2 and 1Dx2.2*subunits into dough indicated that both subunits can lead to yield the dough strength greater than 1Dx2. As both x-type and y-type subunits encoded by the S genome are larger than almost all other known HMW-GSs, we predict that the S genome-encoded HMW-GSs may have an outstanding ability to strengthen the gluten interactions. Based on our results, it will be valuable to further explore the potential values of these novel *Sitopsis* HMW-GS variants in modifying the structure, composition and function of wheat storage proteins. Furthermore, these special S genome-encoded genes and glutenin subunits will be helpful to overcome the bottleneck of poor genetic diversity of *Glu-1* alleles and HMW-GSs in hexaploid wheat. Two approaches are under the way to verify the function of 1Sx subunits. One is to develop wheat transgenic plants which allow the endosperm specific expression of 1Sx alleles; the other is to transfer the 1Sx subunits to tetraploid or hexaploid wheats by the interspecies cross.

## Conclusions

We have identified and characterized six novel HMW-GS variants from three *Aegilops* section *Sitopsis* species. The possession of large molecular weights is unique feature of S genome-encoded HMW-GSs. These *Sitopsis* glutenin subunits with large molecular weights have been resulted from the similar duplication of repetitive domains as those in the subunits 1Dx2.2 and 1Dx2.2*. The S genome-encoded subunits, 1Dx2.2 and 1Dx2.2* have independent origins, although they share similar evolutionary mechanism. Because of their molecular weights much larger than all other known HMW-GSs, these novel *Sitopsis* glutenin subunits can be used as special genetic resources to improve wheat quality breeding.

## Methods

### Plant materials

Sixty-five accessions of *Ae*. *bicornis*, *Ae*. *longissima* and *Ae*. *sharonensis*, kindly provided by USDA-ARS (http://www.ars-grin.gov), were investigated on their HMW-GS profiles by using the SDS-PAGE. Three accessions (CIae 70 of *Ae*. *bicornis*, PI 604122 of *Ae*. *longissima* and PI 584388 of *Ae*. *sharonensis*) with larger HMW-GS combinations were chosen for further cloning and characterization.

### SDS-PAGE and protein sequencing

HMW-GSs of *Ae*. *bicornisAe*. *longissima* and *Ae*. *sharonensis* were extracted from the half of single seed according to Mackie et al. [37]. HMW-GSs from hexaploid wheat cv. Chinese Spring (null, 1Bx7+1By8, 1Dx2+1Dy12) were used as a standard reference for comparison of HMW-GS electrophoretic mobility. Total seed proteins were extracted in the sample buffer containing 0.0625 M Tris-HCl (pH 6.8), 2% (w/v) SDS, 1.5% (w/v) DTT, 10% (v/v) glycerol and 0.1% w/v Coomassie Brilliant Blue R250. The extracts were heated at 95°C for 5 min and centrifuged for 10 min. The supernatant was loaded onto a 10% (w/v) SDS-PAGE gel as described by Shewry et al. [2]. To ensure the experimental accuracy, at least three seeds were analyzed for each accession of these three *Aegilops* section *Sitopsis* species.

After electrophoresis, the proteins were transferred from the gel onto a PVDF (Poly vinylidene fluoride) membrane by western blotting. The membrane was saturated with methanol and stained with 0.1% (w/v) Coomassie BBR250. The HMW subunit bands were then excised for protein sequencing. The N-terminal amino acid sequences of the HMW subunits were determined by GeneCore Bio-Technology company (Shanghai, China) using the PROCISE^TM^494CLC amino acid sequencer of Applied Biosystems.

### Isolations and characterization of *Sitopsis Glu-1* ORFs

Genomic DNAs were extracted from the leaves of two-week-old seedlings by using the CTAB method [38]. To amplify the complete coding regions of HMW-GSs, a pair of primers, P1 (5’-ATGGCTAAGCGGC/TTA/GGTCCTCTTTG-3’) and P2 (5’-CTATCACTGGCTG/AGCCGACAATGCG-3’), were designed according to nucleotide sequences in the conserved 5' or 3' ends of available HMW-GS ORF sequences. The high fidelity LA Taq polymerase (TaKaRa) with GC buffer for GC-rich template was used in the PCR amplification to minimize the errors which were introduced into the sequences. The PCR cycling parameters was 94°C for 5 min, followed by 30 cycles of 94°C for 40 sec, 68°C for 5 min and a final extension step at 72°C for 12 min [39]. PCR products were separated in 1% agarose gels and all DNA fragments were recovered, purified and further ligated into the pMD19-T vector (TaKaRa). The ligated mixtures were transformed into *Escherichia coli* DH5α competent cells. The strategy of primer walking and the nest deletion method [40] were used to obtain the full-length of *Sitopsis Glu-1* ORFs. The DNA sequencing was performed by the Invitrogen Company (Shanghai, China). Each clone was sequenced in two directions, the final nucleotide sequences for each *Glu-1* ORF was determined from the sequencing results of 3 independent clones.

### Bacterial expression of cloned HMW glutenin ORFs

In order to confirm that the novel *Glu-S1* genes expressed proteins that corresponded to those in the grain, we choose 1S^sh^x2.9 and 1S^sh^y2.3 as the representation of *Glu-S1x* and *Glu-S1y* for expressional experiments, as three pairs of x and y-type genes possess highly similar DNA sequences and molecular mass in *Ae*. *bicornisAe*. *longissima* and *Ae*. *sharonensis*. To express of the mature proteins of HMW-GS from *Ae*. *sharonensis*, we designed two pairs of primers for amplifying the mutant ORF from which the sequence coding for signal peptide was removed and introducing appropriate restriction enzyme sites of *Nde*I and *Eco*RI for the mutant ORF to facilitate following cloning and expression. The primers pairs of PET-F1 (CTCACCCATATG GAAGGTGAGGCCTCTGGGCA) and PET-R1 (GGCAATGAATTC CTATCACTGGCTAGCCGACA) were used to amply *1S*^*sh*^*x2.9* while the combination PET-F2 (CTCATCCATATGGAAGGTGAGGCCTCTAGGCA) and PET-R2 (GGCAAT GAATTCCTATCACTGGCTGGCCGACA) were specific for y-type genes of *1S*^*sh*^*y2.3*. PCR conditions for amplifying mutant ORF were identical to those described above except that the template was plasmid DNA purified from the determined clones. After the mutant ORF was cloned into the expression vector pET-30a (Novagen), the recombinant construct was selected to express mature protein in the *E. coli* strain BL21 (DE3). Induction of bacterial expression was performed with 1 mM IPTG for 3 to 5 hours. The expressed proteins were purified by extraction with 50% (v/v) propanol containing 2% (w/v) DTT, and then separated by SDS-PAGE [18].

### Isolations and characterization of the 5’ flanking promoters of HMW-GSs

Two pairs of primers were designed for amplifying promoter regions for both x-type and y-type glutenin subunits in these three *Aegilops* section *Sitopsis* species. The P3 primer (5’-AGGGAAAGACAATGGACATG -3’) was designed from the sequence which was strictly conserved in the 5’ flanking regions of all *Glu-1* loci, whereas the primer P4 (5’-GTCTCGGAGC/T TGC/TTGGTC-3’) and primer P5 (5'-CATCTGGAGCCCCGTGCTC-3’) was derived from the sequence coding for 6 residues (DQQLRD) and (STGLQM), respectively. Each of sequence residues exists only in x-type and y-type HMW-GSs, respectively. The primer combinations P3 + P4 and P3 + P5 are specific for x-type and y-type promoters. The amplification profile was 94°C for 5 min, followed by 35 cycles of 94°C for 40 sec, 60°C for 1 min, and 72°C for 1 min 30 sec, and a final extension step at 72°C for 7 min. PCR products were purified, cloned into pMD19-T, and then sequenced. The final nucleotide sequences for *Glu-1* promoters were also constructed from sequencing at least 3 independent clones.

### Sequence analyses and phylogenetic investigation

The prediction of nucleotide sequences was performed by the DNAman software package (V5. 2. 10; Lynnon Biosoft). Multiple alignments were carried out by using Clustal W (V1.83) for comparisons of either DNA or protein sequences [41]. Alignments were further improved by visual examination and manual adjustment. To characterize the phylogenetic relationship of HMW-GS genes, we compared the S genome-encoded *Glu-1* alleles from these three *Aegliops* section *Sitopsis* species with previously characterized x-type HMW-GSs alleles represented by 1Ax2* (M22208), *1Bx7* (X13927), *1Dx2* (X03346), *1Dx5* (X12928), *1Dx2.1* (AY517724), *1Dx2.2* (AY159367), 1Dx2.2* (AY893508), and also with previously characterized y-type HMW-GSs represented by *1Ay* (EU984508), *1By9* (X61026), *1Dy10* (X12929), *1Dy12* (X03041) and *1Dy10.1* (AY695379). The 5’ flanking promoter sequences, plus the sequences encoding signal peptides and the N-terminal, which is considered as phylogenetically informative [28], were selected to create a multiple alignment by the Clustal W program. All the nucleotide sequences and their alignments have also been listed as supplementary materials (see Additional files 1,2 and 3).The neighbour-joining (NJ) tree was constructed by using the software MEGA 4.02 with the substitute model of Maximum Composite Likelihood [42]. In the NJ analysis, gaps were treated as missing data. The bootstrap values were calculated based on 1000 replications to estimate the topological robustness. For the network analysis, the sites with base substitution or mutation were used to constructed media-joining network in program Network 4.6.0.0 (http://www.fluxus-engineering.com/). The media-joining network was calculated under the default parameters of weights = 10 and epsilon = 0 [43].

## Author contributions

JQT contributed to design and carry out the experiments and wrote the draft; MJ did the cloning of HWM glutenin ORFs; WYM revised the manuscript; LYX and LXJ made contribution to SDS-PAGE analysis and promoter cloning; LZX conducted the analysis of the data and review the manuscript; ZS and ZQZ finished the cloning of promoter, phylogenetic analysis and bacterial expression; ZYL contributed to improve research program. All authors have read and approved the final manuscript.

## Supplementary Material

Additional file 1 **Full alignment of y-type HMW-GS ORF sequences of*Aegilops species*with those encoded by D genome.***Description:* The similarity was showed by different color. The deletions were showed by gaps. Click here for file

Additional file 2 **Full alignment of x-type HMW-GS ORF sequences of*Aegilops species*with those encoded by D genome.***Description:* The similarity was showed by different color. The deletions were showed by gaps. Click here for file

Additional file 3 **Full alignment of promoter sequences of Aegilops species with those of*Glu-A1***, ***Glu-B1*and*Glu-D1*.***Description:* The similarity was showed by different color. The deletions were showed by gaps. Click here for file
